# Synergistic Effects of Hybrid Carbonaceous Fillers of Carbon Fibers and Reduced Graphene Oxides on Enhanced Heat-Dissipation Capability of Polymer Composites

**DOI:** 10.3390/polym12040909

**Published:** 2020-04-14

**Authors:** Yun Seon Lee, Jaesang Yu, Sang Eun Shim, Cheol-Min Yang

**Affiliations:** 1 Institute of Advanced Composite Materials, Korea Institute of Science and Technology (KIST), 92 Chudong-ro, Wanju-gun, Jeonbuk 55324, Korea; t14225@kist.re.kr (Y.S.L.); jamesyu@kist.re.kr (J.Y.); 2 Department of Chemical Engineering, Inha University, 100 Inha-ro, Nam-gu, Incheon 22212, Korea

**Keywords:** mesophase pitch-based carbon fiber, reduced graphene oxide, hybrid carbonaceous filler, thermal conductivity, heat dissipation, polymer composite

## Abstract

In this study, we investigated the synergistic effects of thermally conductive hybrid carbonaceous fillers of mesophase pitch-based carbon fibers (MPCFs) and reduced graphene oxides (rGOs) on the thermal conductivity of polymer matrix composites. Micro-sized MPCFs with different lengths (50 μm, 200 μm, and 6 mm) and nano-sized rGOs were used as the thermally conductive fillers used for the preparation of the heat-dissipation polymer composites. For all MPCF fillers with a different length, the thermal conductivity values of the MPCF/epoxy composites were proportional to the MPCF length and loading amount (0–50 wt%) of MPCFs. For an MPCF:rGO weight ratio of 49:1 (total loading amount of 50 wt%), the thermal conductivity values of MPCF-rGO/epoxy composites loaded with MPCFs of 50 μm, 200 μm, and 6 mm increased from 5.56 to 7.98 W/mK (approximately 44% increase), from 7.36 to 9.80 W/mK (approximately 33% increase), and from 11.53 to 12.58 W/mK (approximately 9% increase) compared to the MPCF/epoxy composites, respectively, indicating the synergistic effect on the thermal conductivity enhancement. The rGOs in the MPCF-rGO/epoxy composites acted as thermal bridges between neighboring MPCFs, resulting in the formation of effective heat transfer pathways. In contrast, the MPCF-rGO/epoxy composites with MPCF:rGO weight ratios of 48:2 and 47:3 decreased the synergistic effect more significantly compared to rGO content of 1 wt%, which is associated with the agglomeration of rGO nanoparticles. The synergistic effect was inversely proportional to the MPCF length. A theoretical approach, the modified Mori-Tanaka model, was used to estimate the thermal conductivity values of the MPCF-rGO/epoxy composites, which were in agreement with the experimentally measured values for MPCF-rGO/epoxy composites loaded with short MPCF lengths of 50 and 200 μm.

## 1. Introduction

In recent years, because of the fact that electronic devices have been downsized, integrated, and functionalized, the total amount of heat generated from devices, during their operation, has remarkably increased. Furthermore, because of the popularization of electric vehicles and the emergence of autonomous vehicles, there is an extensive demand for a more efficient heat-dissipation technology. For this reason, effective dissipation of the generated heat is a major issue from the viewpoint of reliability, durability, and safety of the electronic devices and next-generation vehicles [1,2,3]. Therefore, the way to efficiently transfer and release the heat generated from the devices has become an important issue, which should be considered in the initial design stage of the electronic devices and vehicles [4]. Therefore, various methods to allow heat-dissipation have been suggested in order to overcome this problem. In general, heat-dissipation components can rapidly release heat using metal and ceramic materials with high thermal conductivity and thereby can suppress the temperature rise of parts and products [3,5]. However, metal and ceramic materials have the disadvantages of heaviness, poor formability, and high molding cost, which leads to higher manufacturing cost. Therefore, thermally conductive polymer composite materials, offering the advantages of excellent lightweight, moldability, design freedom, and lower manufacturing cost, have been considered as promising candidates to replace the metal and ceramic materials [1,2,3].

Polymers exhibit extremely low thermal conductivity in the range of 0.1–1.05 W/mK compared to metal and ceramic materials [1,2,3]. Various researches have been carried out to increase the limited thermal conductivity of polymers [1,2,3]. In general, for polymer composites used in applications where electrical insulation is not required, an increased thermal conductivity can be achieved by combining the polymers with thermally conductive carbon-based fillers, such as chopped carbon fibers (CFs), graphite particles, graphene nanoplatelets (GNPs), graphenes, and carbon nanotubes (CNTs) [6,7,8]. The thermal conductivity of polymer composites is strongly influenced not only by the intrinsic thermal conductivity of the polymer matrixes and fillers but also by the shape, size, loading amount, orientation, and dispersion of the fillers in the polymer matrix [5,6,7,8,9]. It is important to design the optimal heat transfer pathways with single or hybrid fillers with high thermal conductivity in composite systems [10,11]. Heavy loading of thermally conductive fillers is required in order to obtain polymer composites with high thermal conductivity, resulting in low mechanical properties and poor processability because of the high viscosity of the polymer-filler mixture. Therefore, to minimize the filler loading amount, it is necessary to design an optimal hybrid filler with synergistic effect. In general, efficient hybridization of micro- and nano-sized fillers can maximize the synergistic effects on the thermal conductivity enhancement of polymer composites, owing to the effective formation of heat transfer pathways [12,13]. 

Mesophase pitch-based carbon fibers (MPCFs) with micro-scaled diameters also exhibit an extremely high thermal conductivity (approximately 500–1000 W/mK), originating from their highly crystalline graphitic structure [14]. Therefore, the application of MPCFs as thermally conductive fillers of polymer composites for high heat-dissipation has recently attracted significant attention from both industry and academic research [15]. According to the measurement of thermal conductivity of single-layer graphene by Barandin et al. the graphene showed an exceptionally high value of 5300 W/mK [16], which is much higher than those of individual multi-walled and single-walled CNTs (~3000 and ~3500 W/mK, respectively) [17,18]. The thermal conductivity of graphene was influenced by concentrations of edge defects, vacancies, and wrinkles as well as grain size and thickness [19]. Reduced graphene oxide (rGO), a representative carbon nanomaterial having a plate shape, has been widely investigated as a thermally conductive filler in polymer composites [20,21]. The rGO sheets, having micro-sized area and nano-sized thickness, have various defect sites because of the synthesis method by oxidation-reduction process, resulting in a low thermal conductivity, contrary to the expectations [22,23].

In this study, we fabricated the epoxy-based composites loaded with the micro-sized MPCFs with three different lengths and hybrid fillers of the micro-sized MPCFs and nano-sized rGOs, and compared their morphologies and heat transfer properties. In addition, we investigated the synergistic effects of hybrid carbonaceous fillers of MPCFs and rGOs with different loading amounts on enhanced heat-dissipation capability of polymer composites. Moreover, the theoretical thermal conductivity values were predicted to confirm the synergistic effect of hybrid carbonaceous fillers.

## 2. Materials and Methods 

### 2.1. Preparation of Thermally Conductive Epoxy-Based Composites

The milled MPCFs (Dialead, K223HM, 11 μm diameter, 50 μm or 200 μm length) and chopped MPCFs (Dialead, K223HE, 11 μm diameter, 6 mm length) were purchased from Mitsubishi Plastic Inc. (Tokyo, Japan). The rGOs (rGO-V50, average lateral dimension more than 20 μm, average thickness of 1.0–1.4 nm) were purchased from Standard Graphene Inc. (Ulsan, Korea). Multi-walled carbon nanotubes (MWCNTs, US4315, average diameter of 50–80 nm, average length of 10–20 μm) were purchased from US Research Nanomaterials Inc. (Houston, TX, USA). More detailed information on their material properties is provided in Table S1 of the Supplementary Material. The epoxy resin (DGEBA, YD-128) and curing agent (Jeffamin D-230) were purchased from Kukdo Chemical Co. Ltd. (Seoul, Korea). 

**Figure 1:**
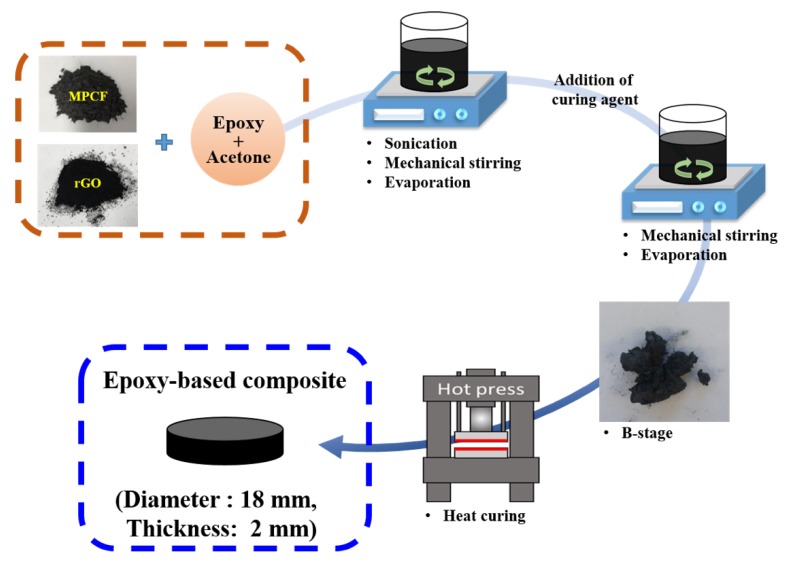
Schematic of fabrication process of epoxy-based composites.

For this study, the composition of the epoxy and curing agent was fixed at a weight ratio of 7:3 [11]. The epoxy resin was diluted with acetone before adding the fillers in order to allow uniform dispersion of thermally conductive fillers within the epoxy resin. The mixture of epoxy resin, acetone, and thermally conductive fillers underwent mechanical stirring for 0.5 h with subsequent bath sonication (40 kHz, 300 W) for 0.5 h. The acetone in the mixture was evaporated in a hot plate at 80 °C for 2 h. Subsequently, the curing agent was added to the mixture, which was mechanically stirred for 0.5 h, and the remaining acetone was completely evaporated. The B-stage composite was cured at 100 °C and 30 MPa for 3 h in a hot press molder that was 18 mm in diameter and 2 mm thick. The epoxy-based composites containing only MPCFs (MPCF length of 50 μm, 200 μm or 6 mm; MPCF loading amounts of 10, 20, 30, 40, and 50 wt%), MPCF-rGO hybrids (MPCF length of 50 μm, 200 μm or 6 mm; MPCF:rGO weight ratios of 50:0, 49:1, 48:2, and 47:3), and MPCF-MWCNT hybrids (MPCF length of 200 μm length; MPCF:MWCNT weight ratios of 50:0, 49:1, 48:2, and 47:3) were prepared. In order to confirm the synergistic effects of MPCF-rGO hybrids on thermal conductivity enhancement, the total loading amounts of only MPCFs and MPCF-rGO hybrid fillers in the epoxy-based composites were adjusted to 50 wt%. The fabrication process of epoxy-based composites is schematically represented in Figure 1.

### 2.2. Characterization of Thermally Conductive Carbonaceous Fillers and Epoxy-Based Composites 

The morphologies of carbonaceous filler powders and their dispersion state in epoxy matrix were characterized using field-emission scanning electron microscopy (FE-SEM, Nova NanoSEM 450, FEI, Hillsboro, OR, USA). The detailed material properties were characterized using micro-Raman spectroscopy with an excitation laser wavelength of 514 nm (inVia Reflecx Raman microscope, Renishaw, Wotton-under-Edge, Gloucestershire, UK). The electrical conductivities of the MPCFs and rGOs fillers were measured using a powder resistivity measurement system (HPRM-M2, Hantech Co. Ltd., Gunpo, Korea) [24]. Sheet conductances of epoxy-based composites were inductively measured with non-contact resistivity measurement system (M-RES-5210D-400, KITEC microelectronic technologie GmbH, Erding, Germany). The measurement principle is based on inducing eddy currents into the test specimen and measuring the resulting magnetic field. Because of the eddy currents, the resulting field depends on the sheet conductance of the test specimen. The advantage of this method is that no electrical contact with the specimen is required. Making reliable and repeatable electrical contact with carbon-based polymer composites is difficult, because the epoxy matrix is non-conductive [25]. The in-plane thermal conductivity values of the fabricated epoxy-based composites were measured using a thermal conductivity measuring instrument (TPS 2500 S, Hot Disk AB, Gothenburg, Sweden) at room temperature, according to standard ISO 22007-2 [26]. Surface temperature monitoring system (GR-STMS-1901, GyoRin Inc., Anyang, Korea) equipped with an infrared thermal camera (FLIR T530, Wilsonville, OR, USA) was used for the evaluation of the surface temperature changes of the fabricated epoxy-based composites during the heating. The specimens (diameter 18 mm, thickness 2 mm) were laid on an isothermal hot plate at a constant temperature of 100 °C. Thermal images of the specimens were recorded for 120 s.

## 3. Results and Discussion

### 3.1. Characterization of Thermally Conductive Carbonaceous Fillers

**Figure 2:**
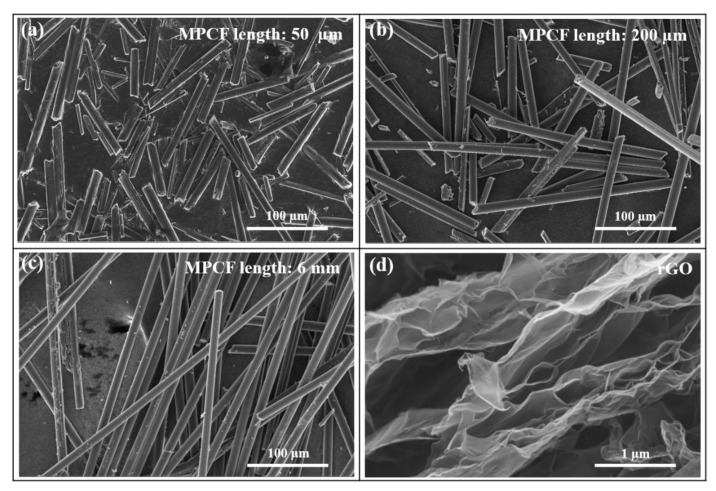
Field-emission scanning electron microscopy (FE-SEM) images of mesophase pitch-based carbon fibers (MPCFs) with different lengths: (**a**) 50 μm, (**b**) 200 μm, and (**c**) 6 mm, and (**d**) reduced graphene oxide (rGO).

In order to confirm the intrinsic properties of thermally conductive carbonaceous fillers used in this study, FE-SEM observation, Raman spectra, and electrical conductivity measurements of MPCFs with different lengths and rGOs were performed. As indicated in the FE-SEM images of the MPCF fillers in Figure 2a–c, the MPCF fillers exhibited a straight fibrous morphology with homogeneous fiber diameters (approximately 11 μm) and different lengths (approximately 50 μm, 200 μm, and 6 mm). Figure 2d shows the typical crumpled morphology of single or few layered graphene sheets with nano-sized thickness and micro-sized lateral area. The distinct shape and size between two carbonaceous fillers are expected to form effective heat transfer pathways in polymer-based composites.

**Figure 3:**
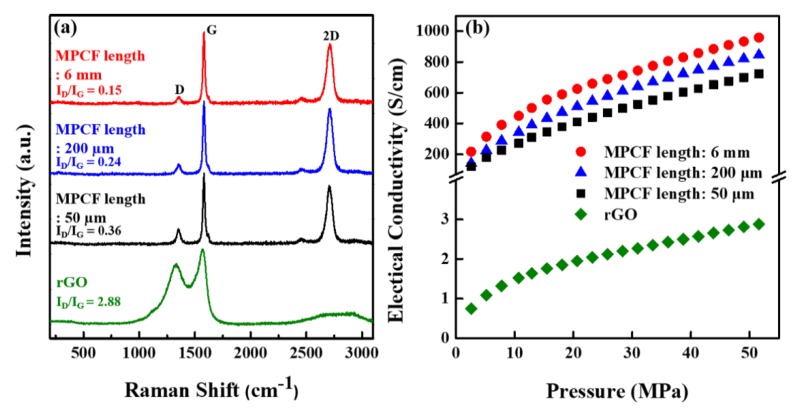
(**a**) Raman spectra and (**b**) electrical conductivity values of MPCF powders with different lengths (50 μm, 200 μm, and 6 mm) and rGO powder.

The structural properties of MPCFs and rGOs were analyzed using Raman spectroscopy, which has been widely used in structural characterization of carbonaceous materials. The *D*-band at approximately 1350 cm^−1^ and *G*-band at approximately 1580 cm^−1^ come from defect-induced disordered and graphitic structure, respectively. Therefore, the ratio between the integrated intensities of the *D*- and *G*-bands (*I_D_/I_G_*) can provide information on the defect concentration of carbonaceous materials. Figure 3a shows the Raman spectra (excited at 514 nm) for the MPCFs with different lengths and rGOs. The Raman spectra of the MPCF samples exhibited extremely low *I_D_/I_G_* ratios, which are similar features to that of graphite with high crystallinity [11]. The *I_D_/I_G_* ratio of the MPCF samples proportionally increased with a decrease in their length, indicating increased concentration of edge defects by shortening of MPCFs during chopping or milling process. In contrast, rGOs had an extremely high *I_D_/I_G_* ratio of 2.88 compared to MPCF samples, because of an increased defect concentration (edges and vacancies) induced from oxidative exfoliation process [22].

Figure 3b shows the electrical conductivity values of the powder samples of MPCF with different lengths and rGO as a function of applied pressure, which was measured with a powder resistivity measurement system. The electrical conductivities of all samples increased with an increase in applied pressure, suggesting that the contact area between adjacent particles of the carbonaceous fillers increases with increasing applied pressure [27]. The MPCF samples had extremely high electrical conductivity values, which is related to the high crystallinity of MPCFs. The electrical conductivity values of MPCF samples proportionally increased with the MPCF length, which is strongly dependent on the *I_D_/I_G_* ratio of MPCF samples. The electrical conductivity values of the rGO powder were much lower than those of the MPCF powders, which is associated with the high defect concentration (high *I_D_/I_G_* ratio value) in the rGO nanosheets. Moreover, the electrical contact resistance at junctions formed between the rGO nanoparticles increased because of an increase of contact points between them. Therefore, the electrical conductivity results of MPCF and rGO powders are in good agreement with their *I_D_/I_G_* ratios in Raman spectra. 

### 3.2. Effect of Mesophase Pitch-Based Carbon Fiber (MPCF) Length on Thermal Conductivity of Epoxy-Based Composites

**Figure 4:**
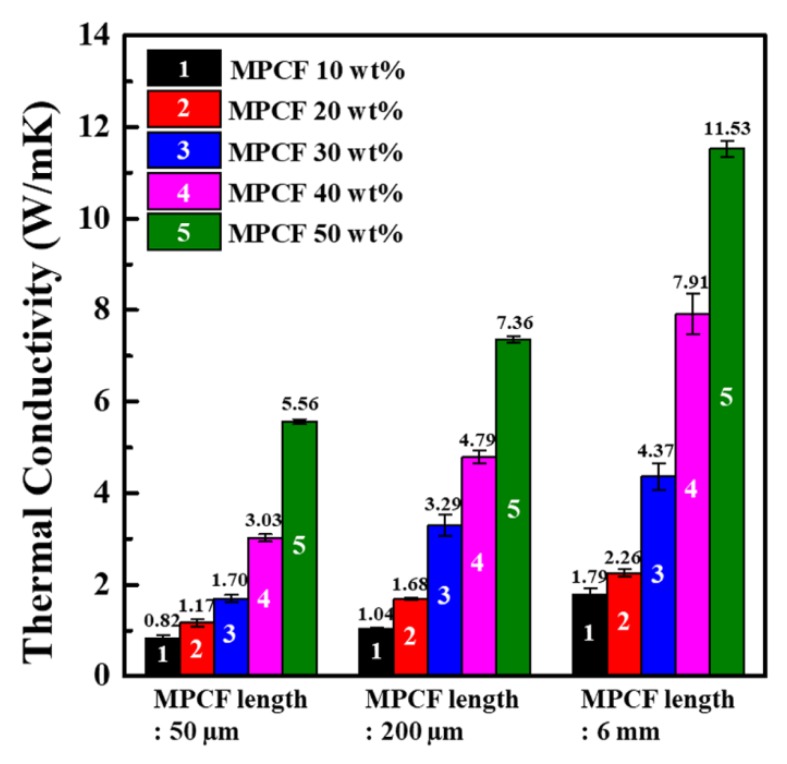
In-plane thermal conductivity values of epoxy-based composites containing MPCFs with different lengths (50 μm, 200 μm, and 6 mm) and different loading amounts (10 to 50 wt%).

To elucidate the effect of the MPCF length on the thermal conductivity, MPCF/epoxy composites were prepared using short-milled MPCFs (50 and 200 μm) and long-chopped MPCFs (6 mm) with different MPCF loading contents in the range of 10–50 wt%, respectively (Figure 4). The thermal conductivity values increased with increasing MPCF loading contents. In particular, at higher MPCF loading contents, the margin of increase in the thermal conductivity was much higher than that at lower MPCF loading contents. The highest thermal conductivity value (11.53 W/mK) was obtained from MPCF/epoxy composite with long-chopped MPCFs (6 mm) of 50 wt%. At all the same MPCF loading contents, the thermal conductivity values of MPCF/epoxy composites proportionally increased with an increase in the MPCF length. The higher thermal conductivity values of the long MPCF/epoxy composite were associated with shortened heat transfer pathways and decreased phonon scattering at the inter-fiber junctions of the longer MPCF network.

**Figure 5:**
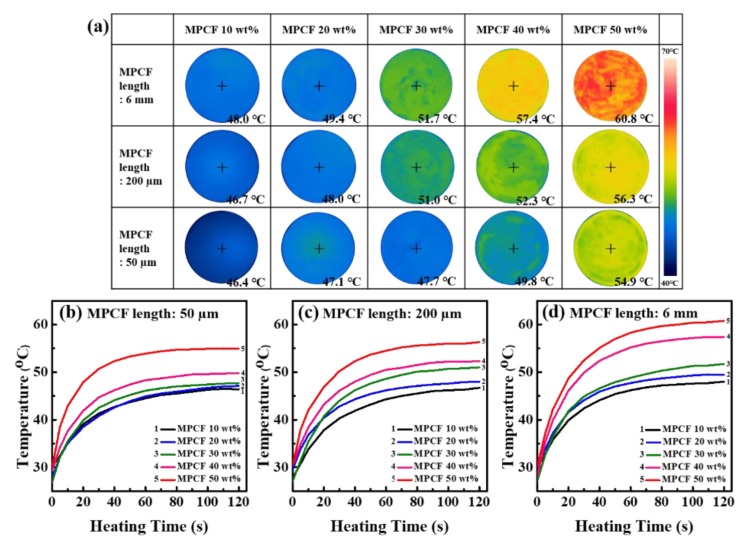
(**a**) Infrared thermal camera images and (**b**) to (**d**) temperature-time profiles of the epoxy-based composites containing MPCFs with different lengths (50 μm, 200 μm, and 6 mm) and different loading amounts (10 to 50 wt%) for transient temperature responses during heating (100 °C, 120 s): (**b**) MPCF 50 μm length, (**c**) MPCF 200 μm length, and (**d**) MPCF 6 mm length.

Figure 5 shows the infrared thermal images and surface temperature variations of MPCF/epoxy composites during heating (100 °C, 120 s), which were observed using the infrared thermal camera. For all MPCF/epoxy composites after heating, the surface temperature increased with increasing MPCF loading contents. At all the same MPCF loading contents, the MPCF/epoxy composites with long-chopped MPCFs (6 mm) reached the highest temperatures. After heating for 120 s, the surface temperature of MPCF/epoxy composite with long-chopped MPCFs (6 mm) reached 60.8 °C, which is higher than MPCF/epoxy composites (50 and 200 μm, 54.9 and 56.3 °C, respectively). These results demonstrated that the long MPCF/epoxy composites form the most effective heat transfer pathways and thereby the heat can be rapidly transferred to the surface by heat conduction.

### 3.3. Synergistic Effects of Hybrid Fillers of MPCF and Reduced Graphene Oxide (rGO) on Thermal Conductivity of Epoxy-Based Composites 

**Figure 6:**
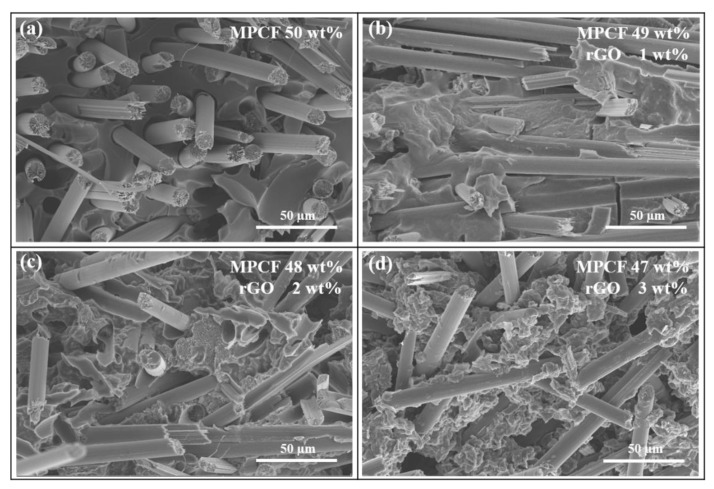
FE-SEM images of epoxy-based composites containing MPCF-rGO hybrid fillers of MPCF length (6 mm) and different rGO contents (0 to 3 wt%). The total loading amounts of only MPCF or MPCF-rGO hybrid fillers in the epoxy-based composites were adjusted to 50 wt%: MPCF:rGO weight ratios of (**a**) 50:0, (**b**) 49:1, (**c**) 48:2, and (**d**) 47:3.

We investigated the synergistic effects of hybrid carbonaceous fillers of micro-sized MPCFs and rGOs with nano-sized thickness and micro-sized lateral area on enhanced heat-dissipation capability of polymer composites. Figure 6 shows the FE-SEM images of the epoxy-based composites loaded with only MPCF and MPCF-rGO hybrid fillers of 50 wt% (MPCF length of 6 mm). The MPCF fillers with straight fibrous morphology were homogeneously dispersed in the epoxy matrix (Figure 6a). For an MPCF:rGO weight ratio of 49:1, the inter-fiber junctions were covered by rGO nanoparticles, resulting in thermal bridging effects of rGOs between neighboring MPCFs (Figure 6b). However, as the rGO content increased to 2 and 3 wt%, the rGO nanoparticles were agglomerated into large particles, resulting in increased thermal contact resistance at the heat transfer pathways of the epoxy-based composites (Figure 6c,d). 

**Figure 7:**
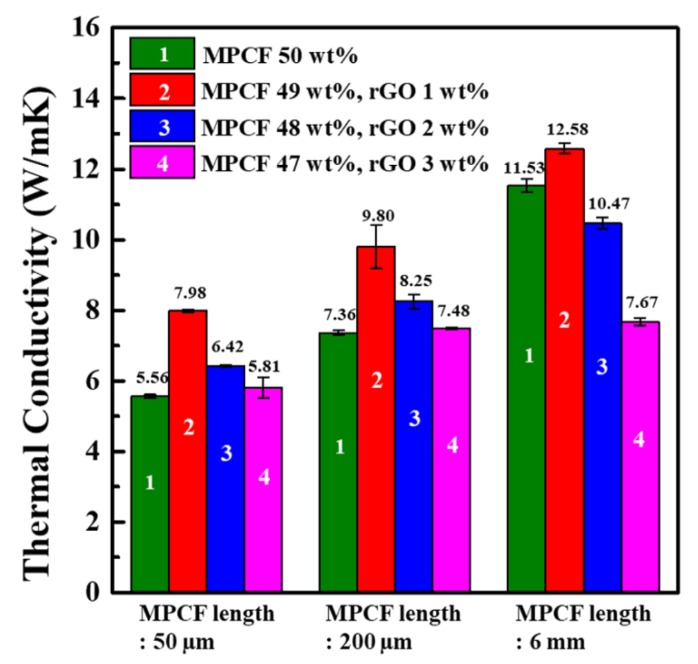
In-plane thermal conductivity values of epoxy-based composites containing MPCF-rGO hybrid fillers of different MPCF lengths (50 μm, 200 μm, and 6 mm) and different rGO contents (0 to 3 wt%).

Figure 7 presents the in-plane thermal conductivity values of epoxy-based composites containing only MPCFs with different lengths and MPCF-rGO hybrid fillers with different weight ratios. The thermal conductivity values of MPCF/epoxy composites at a total filler loading of 50 wt% proportionally increased with an increase in MPCF length as described above. In particular, MPCF/epoxy composite with long-chopped MPCFs (6 mm) of 50 wt% had an extremely high thermal conductivity of 11.53 W/mK compared to those with short-milled MPCFs (50 and 200 μm, 5.56 and 7.36 W/mK, respectively). For an MPCF:rGO weight ratio of 49:1, the thermal conductivity values of MPCF-rGO/epoxy composites loaded with MPCFs of 50 and 200 μm dramatically increased from 5.56 to 7.98 W/mK (approximately ca. 44% increase) and from 7.36 to 9.80 W/mK (approximately ca. 33% increase) compared to the MPCF/epoxy composites, respectively. The rGOs in the MPCF-rGO/epoxy composites might act as thermal bridges between neighboring MPCFs, resulting in the formation of effective heat transfer pathways, as shown in FE-SEM images of MPCF-rGO/epoxy composites (Figure 6b). For MPCF-rGO/epoxy composites with rGO contents of 2 and 3 wt%, the synergistic effect on the thermal conductivity decreased with an increase of rGO content, which could be attributed to an increased thermal contact resistance at junctions formed between the fillers, because of the excessive addition of rGOs with much lower intrinsic thermal conductivity compared to MPCFs, as shown in *I_D_/I_G_* ratios of Raman spectra and electrical conductivity values of MPCFs and rGOs (Figure 6c,d). Moreover, excessive addition of rGOs to the composites could cause the agglomeration of rGO nanoparticles, resulting in a decreased thermal conductivity of the composites (Figure 6c,d). In this study, the synergistic effect on thermal conductivity enhancement in the MPCF-rGO/epoxy composites was maximized at the MPCF:rGO weight ratio of 49:1. Therefore, it is necessary to select an optimal ratio between MPCFs and rGOs to maximize the thermal conductivity of polymer composite.

**Figure 8:**
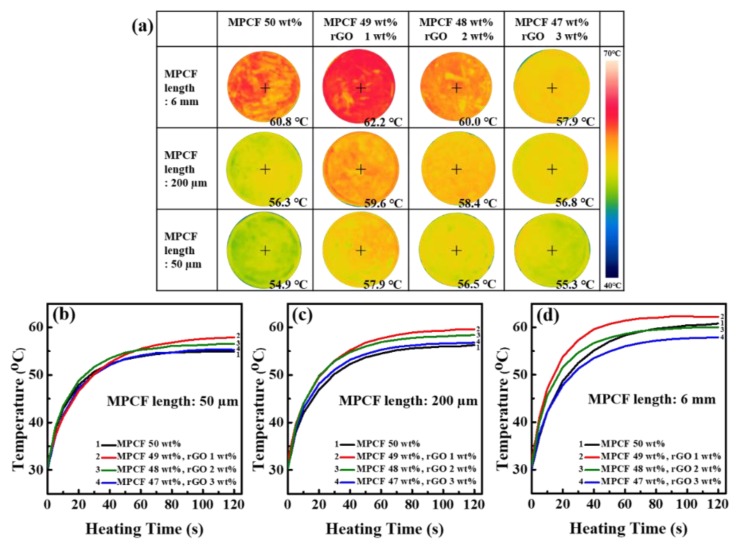
(**a**) Infrared thermal camera images and (**b**) to (**d**) temperature-time profiles of the epoxy-based composites containing MPCF-rGO hybrid fillers with different MPCF lengths (50 μm, 200 μm, and 6 mm) and different rGO loading amounts (0 to 3 wt%) for transient temperature responses during heating (100 °C, 120 s): (**b**) MPCF 50 μm length, (**c**) MPCF 200 μm length, and (**d**) MPCF 6 mm length. The total loading amounts of only MPCF or MPCF-rGO hybrid fillers in the epoxy-based composites were adjusted to 50 wt%.

Figure 8 shows the infrared thermal images and surface temperature variations of MPCF-rGO/epoxy composites. All MPCF-rGO/epoxy composites with an MPCF:rGO weight ratio of 49:1 reached the highest surface temperature after heating (100 °C, 120 s), indicating the maximum synergistic effect of the hybrid carbonaceous filler. In contrast, MPCF-rGO/epoxy composites with long-chopped MPCFs (6 mm) with rGO contents of 2 and 3 wt% showed rather lower surface temperatures compared to the epoxy composite loaded with only MPCFs. This result is associated with the reduced thermal bridging effects of rGOs between neighboring MPCFs because long-chopped MPCFs (50 wt%) with higher aspect ratios easily formed percolation networks for efficient thermal transport in the epoxy-based composites. MPCF-rGO/epoxy composites with short-milled MPCFs (50 and 200 μm) with rGO contents of 2 and 3 wt% had relatively low surface temperatures compared to rGO content of 1 wt%, which is associated with their decreased synergistic effect. Therefore, these results are in good agreement with the thermal conductivity values of the composite samples (Figure 7).

**Figure 9:**
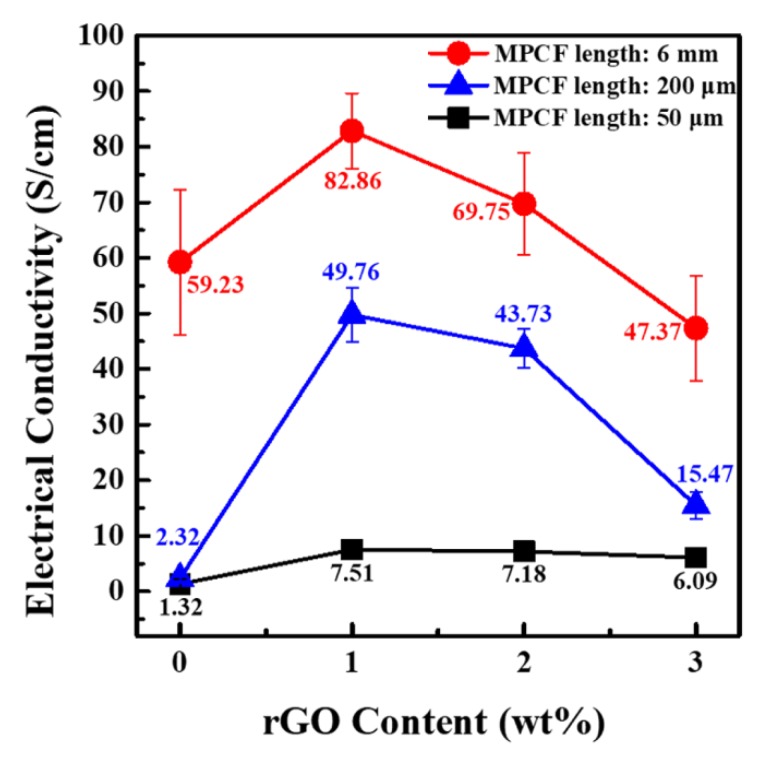
Electrical conductivity values of epoxy-based composites containing MPCF-rGO hybrid fillers of different MPCF lengths (50 μm, 200 μm, and 6 mm) and different rGO contents (0 to 3 wt%). The total loading amounts of MPCFs and rGOs in the epoxy-based composites were adjusted to 50 wt%.

Figure 9 presents the electrical conductivity values of epoxy-based composites containing only MPCFs with different lengths and MPCF-rGO hybrid fillers with different weight ratios. The electrical conductivity values of the composites at a total loading amount of 50 wt% proportionally increased with an increase in MPCF length, which is associated with a low electrical percolation threshold due to higher aspect ratio of MPCFs [28]. For all MPCF fillers with different lengths, the maximum synergistic effects on electrical conductivity were observed at an MPCF:rGO weight ratio of 49:1. The electrical conductivity values of MPCF/epoxy and MPCF-rGO/epoxy composites exhibited a similar trend with their thermal conductivity behavior.

In order to compare the synergistic effects between MPCF-rGO and MPCF-MWCNT composites, MPCF-MWCNT/epoxy composites were prepared with MPCF (200 μm length) and MWCNT in the MPCF:MWCNT weight ratios of 50:0, 49:1, 48:2, and 47:3 and their thermal conductivity values were measured. As a result, the synergistic effect of the MPCF-rGO composites was much more pronounced compared with that of the MPCF-MWCNT composites (Figure S1 of the Supplementary Material). MWCNTs, which have the shapes of thin fibers (diameter of 50–80 nm), have smaller contact areas between the MPCFs and MWCNTs compared with the larger contact areas of the 2D-shaped rGOs. Therefore, the hybridization of MPCF-rGO fillers could maximize the synergistic effects on the thermal conductivity enhancement of polymer composites compared with MPCF-MWCNT hybridization, owing to the effective formation of thermal bridges.

### 3.4. Theoretical Approach of Thermal Conductivity in the MPCF-rGO/Epoxy Composites

In this study, the modified Mori-Tanaka theoretical approach developed in a previous study [29,30] was used to estimate the thermal conductivities of polymer matrix composites containing MPCFs and rGOs. The Mori-Tanaka method (MTM) considers a single ellipsoidal heterogeneity embedded within an infinite homogeneous matrix domain subjected to constant far-field heat flux, as applied to steady-state heat conduction problems. The MTM differs from the Eshelby method, which suggests that the thermal gradient field is not perturbed by the existence of heterogeneities within a matrix. On the contrary, MTM uses the continuum averaged heat flux vector (*q*) and temperature gradient (∇T) to predict the effective thermal conductivity tensor for the composite. The heat flow in a composite may be characterized in terms of the far-field applied heat flux vector (*q*), i.e.,

(1) $$q=-\bar{K}\cdot \nabla T$$

where $\bar{K}$ is the effective second-rank thermal conductivity tensor and ∇T is the continuum averaged temperature gradient. Similar to the classical Eshelby solution for linear elasticity, where the strain field inside each heterogeneity is constant, the resulting temperature gradient inside each heterogeneity is also constant when calculating the effective thermal properties. For a composite with a matrix phase (0) and a nano-reinforcement phase (1), the second-rank effective thermal conductivity tensor ($\bar{K}$) can be expressed as follows:

(2) $$\bar{K}=K_{\left(0\right)}\cdot \left\{I+c_{1}\left(S_{\left(1\right)}-I\right)\cdot {\left(A_{\left(1\right)}-S_{\left(1\right)}\right)}^{-1}\right\}\cdot {\left\{I+c_{1}S_{\left(1\right)}\cdot {\left(A_{\left(1\right)}-S_{\left(1\right)}\right)}^{-1}\right\}}^{-1}$$

(3) $$A_{\left(1\right)}={\left(K_{\left(0\right)}-K_{\left(1\right)}\right)}^{-1}\cdot K_{\left(0\right)}$$

where *A*_(1)_ is the second-rank thermal gradient concentration tensor for heterogeneity, *K*_(0)_ and *K*_(1)_ are the second-rank thermal conductivity tensors for the matrix and heterogeneity, *c*_1_ is the heterogeneity volume fraction, *S*_(1)_ is the second-rank Eshelby tensor for heterogeneity, *I* is the second-rank identity tensor, and a middle dot is used to denote the tensor single dot product. The Eshelby tensor (*S*_(1)_) accounts for the influence of the aspect ratio/geometry of the heterogeneity on the local temperature field. 

The MTM may be extended to composites with multiple distinct heterogeneities (i.e., fibers, spheres, platelets, voids, etc.,) using the multi-inclusion and multi-phase composite models. Suppose that the matrix contains *m* distinct types of ellipsoidal heterogeneities (*p* = 1, 2..., *m*) each consisting of *n_p_* layers (*α_p_* = 1, 2..., *n_p_*; *p* = 1, 2..., *m*) [29,30]. Each type of heterogeneity has distinct thermal properties, shapes, and orientation distributions. In this case, the effective thermal conductivity tensor, $\bar{K}$, can be expressed as follows:

(4) $$\bar{K}=K_{\left(0\right)}\cdot \{I+\overset{m}{\underset{p=1}{\sum }}[\overset{n_{p}}{\underset{\alpha_{p}=1}{\sum }}c_{{\left(p\right)}_{\alpha_{p}}}\left(S_{\left(p\right)}-I\right)\cdot \left(A_{\left(p\right)}{}^{\left(\alpha_{p}\right)}-S_{\left(p\right)}{)}^{-1}\right]\}\cdot \{I+\overset{m}{\underset{p=1}{\sum }}{\left[\overset{n_{p}}{\underset{\alpha_{p}=1}{\sum }}c_{{\left(p\right)}_{\alpha_{p}}}S_{\left(p\right)}\cdot \left(A_{\left(p\right)}{}^{\left(\alpha_{p}\right)}-S_{\left(p\right)}{)}^{-1}\right]\right\}}^{-1}$$

(5) $$A_{\left(p\right)}{}^{\left(\alpha_{p}\right)}={\left(K_{\left(0\right)}-K_{\left(p\right)}{}^{\left(\alpha_{p}\right)}\right)}^{-1}\cdot K_{\left(0\right)}$$

where $A_{\left(p\right)}{}^{\left(\alpha_{p}\right)}$ is the second-rank temperature gradient concentration tensor for the *α_p_*^th^ layer of the *p*^th^ heterogeneity (*α_p_* = 1, 2..., *n_p_*; *p* = 1, 2 …, *m*), $K_{\left(p\right)}{}^{\left(\alpha_{p}\right)}$ is the second-rank thermal conductivity tensor for the *α_p_*^th^ layer of the *p*^th^ heterogeneity, $c_{{\left(p\right)}_{\alpha_{p}}}$ is the volume fraction of the *α_p_*^th^ layer of the *p*^th^ heterogeneity, and *S*_(*p*)_ is the second-rank Eshelby tensor common to the *p*^th^ heterogeneity and all of its layers. By these estimations, MPCFs dimensions of 50, 200, and 6000 μm (6 mm) were used in order to compare the values obtained from experiments and theoretical calculations. The values of density and thermal conductivity for an epoxy, MPCFs, and rGOs used for theoretical approaches were 1.150 (measured using the Archimedes’ principle), 0.845, and 0.161 g/cc (measured using a powder resistivity measurement system) and 0.23 (measured using a thermal conductivity measuring instrument), 550.0 [31], and 776.0 W/mK [32], respectively.

**Figure 10:**
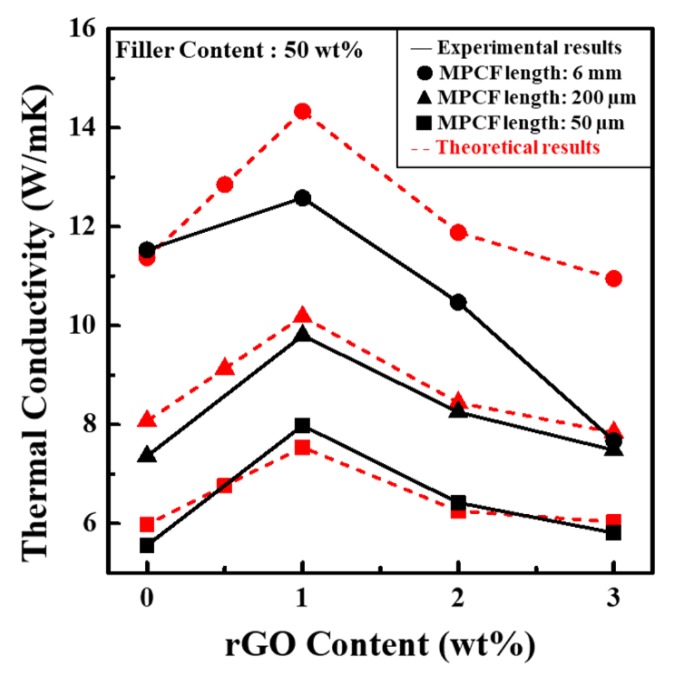
Experimental and theoretical thermal conductivity values of epoxy-based composites containing MPCF-rGO hybrid fillers of different MPCF lengths (50 μm, 200 μm, and 6 mm) and different rGO contents (0 to 3 wt%).

Figure 10 shows the experimental and theoretical thermal conductivity values for the MPCF-rGO/epoxy composites. The estimated thermal conductivity values of MPCF-rGO/epoxy composites increased as the contents of rGOs increased up to 1 wt%, compared to those of MPCF/epoxy composites. For the MPCF-rGO/epoxy composites with rGO contents of 2 and 3 wt%, the estimated thermal conductivity values decreased with an increase of rGO content, indicating a decreased synergistic effect on the thermal conductivity due to the anti-bridging effects by increased agglomeration rates of rGOs, as discussed in the previous section. Therefore, according to our theoretical calculation, MPCF-rGO/epoxy composites with an MPCF:rGO weight ratio of 49:1 had the highest thermal conductivity value for the range of composites with rGO contents of 0–3 wt%, which is supported by the experimentally measured value for the MPCF-rGO/epoxy composite loaded with MPCF (length of 50 μm, 49.5 wt%) and rGO (0.5 wt%) (Figure S2 of the Supplementary Material). In order to capture this trend in the modeling, the agglomeration rates used for rGO contents of 2 and 3 wt% were 32 and 38 vf% out of 100 vf% rGOs in these estimations, respectively. These values can be accurately determined by the statistical data obtained from FE-SEM observations. The estimated thermal conductivity values of MPCF-rGO/epoxy composites with short-milled MPCFs (50 and 200 μm) were reasonably in accordance with the experimentally measured values, as shown in Figure 10. On the other hand, for MPCF-rGO/epoxy composites with long-chopped MPCFs (6 mm), the theoretically estimated thermal conductivity values significantly deviated from experimentally measured values, which might be associated with the accelerated agglomeration of rGO nanoparticles in epoxy-based composites. This might be because long MPCF networks prevent rGO nanoparticles from homogeneous dispersion.

## 4. Conclusions

In this study, the synergistic effects of hybrid carbonaceous fillers of MPCFs and rGOs on the enhanced heat-dissipation capability of polymer composites were investigated. For all micro-sized MPCF fillers with different length (50 μm, 200 μm, and 6 mm), the thermal conductivity values of MPCF/epoxy composites were proportional to the loading amount and fiber length of MPCFs. In particular, the maximum synergistic effects on thermal conductivity enhancement were observed for MPCF-rGO/epoxy composites with an MPCF:rGO weight ratio of 49:1, which is associated with the effective formation of heat transfer pathways, compared to those of 48:2 and 47:3. Moreover, the synergistic effect was inversely proportional to the MPCF length. Therefore, the nano-sized rGO nanoparticles of optimal content in the MPCF-rGO/epoxy composites acted as thermal bridges between neighboring MPCFs, while excessive addition of the rGO nanoparticles induced anti-bridging effects in the MPCF networks by agglomeration of rGOs. For MPCF-rGO/epoxy composites loaded with short MPCF lengths of 50 and 200 μm, the thermal conductivity values estimated by the modified Mori-Tanaka theoretical approach showed that the results obtained from modeling were in agreement with those obtained from experiments, which can be explained by reasonable concepts of bridging and anti-bridging effects of the nano-sized fillers in the heat transfer pathways of polymer composites.

## Supplementary Materials

The following are available online at https://www.mdpi.com/2073-4360/12/4/909/s1. Table S1: Summary of basic properties of carbonaceous fillers; Figure S1: In-plane thermal conductivity values of epoxy-based composites containing MPCF-rGO and MPCF-MWCNT hybrid fillers (MPCF length of 50 μm and different rGO or MWCNT contents of 0 to 3 wt%); Figure S2. Experimental and theoretical thermal conductivity values of epoxy-based composites containing MPCF-rGO hybrid fillers of MPCF length of 50 μm and different rGO contents (MPCF:rGO weight ratios of 50:0, 49.5:0.5, 49:1, 48:2, and 47:3).
