# Supplementary material for: Synergistic Effects of Hybrid Carbonaceous Fillers of Carbon Fibers and Reduced Graphene Oxides on Enhanced Heat-Dissipation Capability of Polymer Composites
Source: Polymers (Basel). 2020 Apr 14;12(4):909. doi: 10.3390/polym12040909 (PMC7240675; doi:10.3390/polym12040909)
Supplement: Supplementary file 1 [file polymers-12-00909-s001.pdf]

polymers

MDPI

Supplementary Data

Article

Synergistic Effects of Hybrid Carbonaceous Fillers of Carbon Fibers and Reduced Graphene Oxides on Enhanced Heat-Dissipation Capability of Polymer Composites

Yun Seon Lee[1,2], Jaesang Yu[1], Sang Eun Shim[2,*] and Cheol-Min Yang[1,*]

[1] Institute of Advanced Composite Materials, Korea Institute of Science and Technology (KIST), 92 Chudong-ro, Wanju-gun, Jeonbuk 55324, Republic of Korea; t14225@kist.re.kr (Y.S.L.); jamesyu@kist.re.kr (J.Y.)

[2] Department of Chemical Engineering, Inha University, 100 Inha-ro, Nam-gu, Incheon 22212, Republic of Korea

* Correspondence: seshim@inha.ac.kr (S.E.S.); cmyang1119@kist.re.kr (C.-M.Y.)

Table S1. Summary of basic properties of carbonaceous fillers.

Material	Mesophase Pitch-Based Carbon Fiber (MPCF)			Reduced Graphene Oxide (rGO)	Multi-Walled Carbon Nanotube (MWCNT)
Model	K223HE	K223HM		V50	US4315
Physical size	6 mm (length of fiber)	200 μm (length of fiber)	50 μm (length of fiber)	1.0 ~ 1.4 nm (average through-plane dimension)	50 ~ 80 nm (outside diameter)
	11 μm (diameter of mono-filament)			≥ 20 μm (average lateral dimension)	10 ~ 20 μm (length)
Carbon content	≥ 99 %			76 ~ 82 %	97 %
Thermal conductivity	550 W/mK (fiber axial direction)			-	-
Electrical conductivity (powder resistance)	959 S/cm	845 S/cm	724 S/cm	2.88 S/cm	-
I_D/I_G ratio (Raman)	0.15	0.24	0.36	2.88	-
Specific surface area	-			500 ~ 780 m^2/g	40 m^2/g

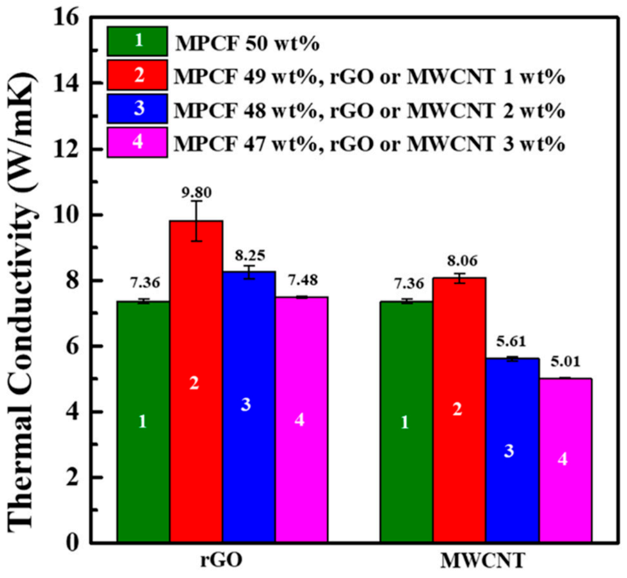

Figure S1. In-plane thermal conductivity values of epoxy-based composites containing MPCF-rGO and MPCF-MWCNT hybrid fillers (MPCF length of 50 μm and different rGO or MWCNT contents of 0 to 3 wt%).

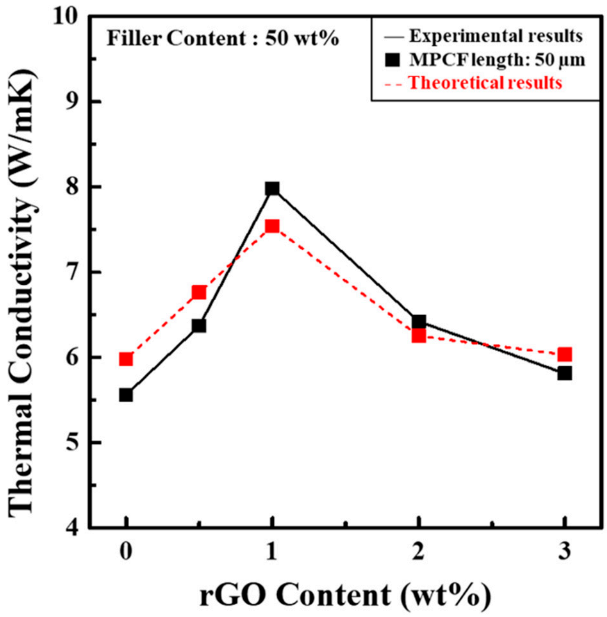

Figure S2. Experimental and theoretical thermal conductivity values of epoxy-based composites containing MPCF-rGO hybrid fillers of MPCF length of 50 μm and different rGO contents (MPCF:rGO weight ratios of 50:0, 49.5:0.5, 49:1, 48:2, and 47:3).
